# Development of the SPARK family member web pages to improve symptom management for pediatric patients receiving cancer treatments

**DOI:** 10.1186/s12885-020-07433-9

**Published:** 2020-09-25

**Authors:** Cody Z. Watling, Clodagh McCarthy, Alexandra Theodorakidis, Sadie Cook, Emily Vettese, Tal Schechter, Hanan Abubeker, L. Lee Dupuis, Lillian Sung

**Affiliations:** 1 Program in Child Health Evaluative Sciences, Peter Gilgan Centre for Research and Learning, The Hospital for Sick Children, 686 Bay Street, Toronto, Ontario Canada; 2 Canadian Cancer Society, 55 St Clair St W., Toronto, Ontario Canada; 3 AboutKidsHealth, The Hospital for Sick Children, 555 University Avenue, Toronto, Ontario Canada; 4 Department of Pediatrics, Division of Haematology/Oncology, The Hospital of Sick Children and Faculty of Medicine, University of Toronto, 555 University Avenue, Toronto, Ontario Canada; 5 Research Institute and Department of Pharmacy, The Hospital for Sick Children, Leslie Dan Faculty of Pharmacy, University of Toronto, 555 University Avenue, Toronto, Ontario Canada; 6 Division of Haematology/Oncology, Department of Paediatrics, The Hospital for Sick Children, 555 University Avenue, Toronto, Ontario Canada

**Keywords:** Pediatric cancer, Supportive care, Website development, Family member, Symptom screening, Education

## Abstract

**Background:**

Supportive care Prioritization, Assessment and Recommendations for Kids (SPARK) is a web-based application that facilitates symptom screening and access to supportive care clinical practice guidelines (CPGs) for children and adolescents receiving cancer treatments. Objective was to develop SPARK family member web pages for pediatric patient family members accessing: (1) proxy symptom screening and symptom reports, and (2) care recommendations for symptom management based on CPGs.

**Methods:**

SPARK family member web pages were developed and included access to symptom screening and care recommendations sections. Care recommendations for fatigue and mucositis were created. These were iteratively refined based upon cognitive interviews with English-speaking family members ≥16 years of age until less than two participants incorrectly understood sections as adjudicated by two independent raters.

**Results:**

A total of 100 family members were enrolled who evaluated the SPARK family member web pages (*n* = 40), fatigue care recommendation (*n* = 30) and mucositis prevention care recommendation (*n* = 30). Among the last 10 participants, none said that the SPARK family member web pages were hard or very hard to use, one incorrectly understood one web page, none said either care recommendation was hard to understand and none were incorrect in their understanding of the care recommendations.

**Conclusions:**

We successfully developed SPARK web pages for use by family members of pediatric patients receiving cancer treatments. We also developed a process for translating CPG recommendations designed for healthcare professionals to lay language. The utility of SPARK family member web pages after clinical implementation could be a focus for future research.

## Background

Most children who are diagnosed with cancer in high income countries will survive, although many will require intensive therapies. These therapies can be associated with severely bothersome symptoms including nausea, mouth sores, fatigue and pain [1–3]. Systematic symptom screening linked to symptom management clinical practice guidelines (CPGs) is needed to optimize care for children receiving cancer treatment and to improve their quality of life.

The Symptom Screening in Pediatrics Tool (SSPedi) was developed to facilitate symptom screening for children receiving cancer treatment [4–7]. This tool asks children how bothered they are by 15 symptoms either yesterday or today [4]. It has been validated for self-report by children 8–18 years and for proxy-report by a family member [7, 8]. To link the identification of symptoms to recommendations for their management, Supportive care Prioritization, Assessment and Recommendations for Kids (SPARK) was developed. SPARK is a web-based application that consists of a symptom screening component centered on SSPedi and a supportive care CPG component. SPARK is intended for use by pediatric patients, family members and healthcare providers. We previously described the initial development of the SPARK web pages for use by pediatric patients [9, 10].

Family members are key advocates for pediatric patients receiving cancer treatments [11]. We envisioned that family members may use SPARK to proxy-report symptoms or to learn about evidence-based recommendations for symptom management. While proxy-reporting of patient-reported outcomes is a common approach in pediatric studies [12], few have focused on the development of an approach appropriate for clinical implementation. Similarly, while much effort has been devoted to creating CPGs for healthcare professionals [13, 14], little effort has been devoted to translating CPG recommendations for use by key stakeholders such as family members.

Consequently, our objective was to develop SPARK family member web pages accessing: (1) proxy-report symptom screening and symptom reports, and (2) care recommendations for symptom management.

## Methods

SPARK is intended for use by pediatric patients, family members and healthcare providers. This study focused on web pages aimed at family members of pediatric patients receiving cancer treatments, including patients without cancer undergoing hematopoietic stem cell transplantation (HSCT). The SPARK family member web pages were envisioned to have two main functions. First, the web pages would allow family members to track their child’s SSPedi symptom scores. Second, the web pages would contain evidence-based care recommendations that provide guidance on symptom management. This qualitative study used think aloud techniques, cognitive interviewing and iterative revision to develop SPARK web pages aimed at family members of pediatric patients receiving cancer treatments. This study was conducted at a single institution, The Hospital for Sick Children (SickKids), Toronto, Canada. It was approved by the SickKids’ Research Ethics Board and all participants provided informed consent.

To develop SPARK family member web pages, this research consisted of two distinct components. First, we developed the SPARK family member web pages themselves, including the family member home page and access to proxy-SSPedi and care recommendation sections. Second, we developed two family member care recommendations to illustrate examples of the process. For this study, we focused on care recommendations directed at the management of fatigue and prevention of mucositis (termed mouth sores for the SPARK family member web pages).

### Participants

Eligible participants were identified by their healthcare team and were recruited consecutively from the inpatient wards and outpatient clinics. Eligible family member participants included parents, grandparents, aunts, uncles and others who provide care for children and adolescents with cancer and pediatric HSCT recipients (≤ 18 years of age). Participants had to be ≥16 years of age, understand English and be free of cognitive, visual or hearing limitations that would prevent use of SPARK, as judged by a member of the patient’s healthcare team. One family member per patient could be interviewed once regarding either the SPARK family member web pages or one family member care recommendation.

### General procedures

Participants first completed a demographic questionnaire. Next, a participant could review the SPARK web pages alone, the SPARK web pages plus one care recommendation or up to two care recommendations alone depending on the study recruitment timeline. If the SPARK web pages and a care recommendation were reviewed, the participant was first led through the web pages until the care recommendations landing page was reached. Then, the participant was asked to open a specific care recommendation.

All interviews were conducted by trained clinical research associates with experience in cognitive interviewing. Two research associates conducted all interviews together. One interviewer engaged with the participant while the second took field notes. The interviews were not audio-recorded.

### Development of the SPARK family member web pages

The SPARK family member web pages evaluated are shown in Additional file 1 and were: (1) SPARK landing page (for patients, family member and healthcare providers), (2) family member home page (allows family members to access proxy-SSPedi or care recommendations), (3) single SSPedi administration report (shows results of a single administration of all 15 symptoms included in SSPedi), (4) specific symptom longitudinal report (shows a single SSPedi symptom degree of bother over time on a line graph), and (5) family member care recommendation landing page (lists available care recommendations translated for family members).

Family member participants were initially given time to explore the SPARK website freely. Throughout this exploration, participants were encouraged to think aloud [15]. Then, interviewers used a semi-structured interview guide to solicit information regarding the interviewees’ preferences and understanding of specific elements of the web pages. The interviewer started at the “SPARK Landing Page” and asked the participant about their overall understanding of it. The bottom of the landing page contains an explanation of SPARK and SSPedi; understanding of this section was also examined. Then, participants were asked to access the “Family Member Home Page”, which was accomplished by clicking on the family member icon. Upon entering this section, there are two icons: (1) “Track Your Child’s Symptoms”, and (2) “See Care Recommendations”. We evaluated their overall understanding of this page, what could be done on it and how they would track their child’s symptoms.

Next, we evaluated understanding of the “Single SSPedi Administration Report”, which shows degree of bother for all 15 symptoms on a bar graph. We evaluated overall understanding of the report and asked them to interpret the degree of bother for a single symptom. The second report evaluated was the “Specific Symptom Longitudinal Report”, which shows a single symptom over time on a line graph. We determined if the participant could navigate to the report, whether they understood a specific symptom over time, and if they could interpret the degree of bother for a specific symptom on a specific date.

Finally, we evaluated the “Care Recommendation Landing Page”. This page shows all available care recommendations formatted for family members. We evaluated whether the participant understood the landing page overall including whether they could navigate to a specific care recommendation.

Outcomes used to evaluate the SPARK family member web pages were as follows. In terms of the web pages overall, at the conclusion of web pages evaluation, we asked about its overall ease of use on a 5-point Likert scale ranging from 1 = ‘very hard’ to 5 = ‘very easy’. In terms of evaluation of each web page, two outcomes were used to decide whether the SPARK family member web pages were considered satisfactory or required further modification. First, we determined whether the participant was incorrect in their understanding of each of the evaluated web pages as rated by the two clinical research associates participating in the interview on a 4-point Likert scale ranging from 1 = “completely incorrect” to 4 = “completely correct”. The two research associates independently rated understanding during the interview and compared scores upon its completion. If initial ratings disagreed, the final rating was decided by consensus. Second, we considered qualitative comments from the participants and their suggestions to improve the web pages.

### Development of the family member care recommendations

CPGs eligible for translation to lay language appropriate for family members were those focused on supportive care (excluding treatment and late effects CPGs) and those endorsed by the Children’s Oncology Group [16]. For this study, we focused on describing the development and evaluation of the “Managing Fatigue” and “Preventing Mouth Sores” care recommendations that were based upon CPGs developed by the Pediatric Oncology Group of Ontario [17, 18]. To guide development, we convened a panel consisting of a pediatric oncology pharmacist (LD) with CPG expertise, a pediatric oncologist (LS) with CPG expertise and experts in health literacy (CMcC and AT). The clinical research associate interviewers were also members of this panel (CZW, SC, EV and HA).

In creating the draft family member care recommendations, the structure and meaning of the source CPG recommendations were retained. For example, strong recommendations were conveyed using the verb “will”, while weak recommendations were conveyed using the verb “may”. A style guide was developed to ensure consistency. The panel met in person to review and revise the initial draft of the family member care recommendation. Each section was reviewed for fidelity to the source CPG and literacy level. Once the panel was satisfied with the initial version of the care recommendation, it was presented to family member participants for evaluation.

Participants were given time to review the entire care recommendation. Then, each section of the care recommendation was presented and evaluated separately. First, the participant read the section (either silently or out loud, whichever they preferred). Second, the interviewer asked the participant to explain the meaning of the section using a semi-structured interview and probed when understanding was unclear. Third, the interviewer asked the participant to rate how easy or hard the section was to understand on a 5-point Likert scale ranging from 1 = “very hard” to 5 = “very easy”. If participants rated understandability as hard or very hard, the interviewers asked for suggestions to improve understandability.

Three outcomes were used to decide whether a care recommendation required modification or was considered satisfactory. First, we considered the number of participants who rated each section as hard or very hard to understand. Second, we determined whether the participant was incorrect in their understanding of each of the evaluated sections as rated individually by two clinical research associates on a 4-point Likert scale ranging from 1 = “completely incorrect” to 4 = “completely correct”. Similar to evaluation of the SPARK family member web pages, each clinical research associate performed ratings during the interview, compared scores after the interview and arrived at consensus scores in the event of disagreement. Third, we also considered the qualitative comments and participants’ suggestions to improve the care recommendation.

### Modification of the SPARK family member web pages and care recommendations

The study team (for the SPARK family member web pages) or the development panel (for the family member care recommendations) met after each group of five evaluable interviews were completed to decide whether the content required modification. An interview was considered inevaluable if two clinical research associates were not present during an interview or if participants did not complete the interview. Modification was required when two or more participants among the last cohort of 10 participants were completely or mostly incorrect in their understanding of a web page or care recommendation section, or qualitative comments suggested changes were required. For care recommendations, modification was also required if two or more participants among the last cohort of 10 participants found a section hard or very hard to understand. We planned to enroll 20 to 40 participants to evaluate the SPARK family member web pages and each care recommendation (maximum of 120 participants).

## Results

**Fig. 1:**
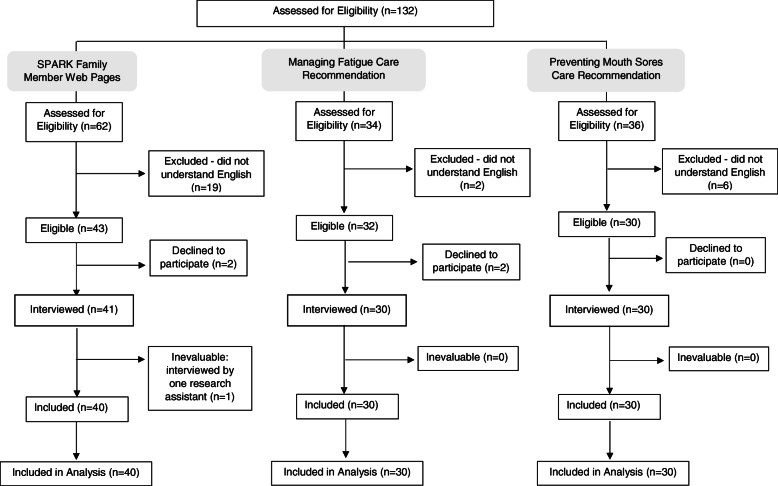
Flow diagram of family member identification and participation. Figure shows SPARK family member web pages evaluated. Arrows shows order of evaluation. Abbreviation: SPARK – Supportive care Prioritization, Assessment and Recommendations for Kids

A total of 100 family members were interviewed. Figure 1 shows the flow diagram of participant eligibility stratified by the component evaluated. Table 1 illustrates the participant and patient demographic characteristics.

**Table 1:** Demographic characteristics of family member participants and their children

	SPARK Family Member Web Pages (***n*** = 40)	Fatigue Care Recommendation (***n*** = 30)	Mouth Sores Care Recommendation (***n*** = 30)
**Family Member**			
Relationship to Patient, n (%)			
Mother	23 (58%)	22 (73%)	26 (87%)
Father	14 (35%)	7 (23%)	4 (13%)
Other	3 (8%)	1 (3%)	0 (0%)
Age in Years, Median (Range)	45 (20–64)	40 (22–62)	40 (28–51)
Male Sex, n (%)	14 (35%)	7 (23%)	4 (13%)
English First Language, n (%)	37 (93%)	21 (70%)	21 (70%)
Race, n (%)			
White	26 (65%)	12 (40%)	16 (53%)
Asian	4 (10%)	7 (23%)	4 (13%)
Black	0 (0%)	3 (10%)	2 (7%)
Mixed ethnicity or other	3 (8%)	7 (23%)	8 (27%)
Missing or unknown	7 (18%)	1 (3%)	0 (0%)
Education, n (%)			
High school	10 (25%)	4 (13%)	6 (20%)
College or university	30 (75%)	26 (87%)	24 (80%)
Household Income, n (%)			
≤ $39,999	5 (13%)	5 (17%)	5 (17%)
$40,000–$79,999	11 (28%)	7 (23%)	8 (27%)
≥ $80,000	23 (58%)	17 (57%)	14 (14%)
Missing	1 (3%)	1 (3%)	3 (10%)
Employment Status, n (%)			
Full-time	16 (40%)	10 (33%)	11 (37%)
Part-time	7 (18%)	2 (7%)	3 (10%)
Stay at home or on leave	16 (40%)	17 (57%)	16 (53%)
Student or missing	1 (3%)	1 (3%)	0 (0%)
Marital Status, n (%)			
Married or common law	27 (68%)	25 (83%)	23 (77%)
Separated or divorced	11 (28%)	3 (10%)	5 (17%)
Single or never married	2 (5%)	2 (7%)	2 (7%)
**Patient**			
Age in Years, Median (Range)	10 (0–18)	12 (1–17)	11 (3–17)
Male Sex, n (%)	24 (60%)	21 (70%)	18 (60%)
English as First Language, n (%)	37 (93%)	25 (83%)	24 (80%)
Diagnosis, n (%)			
Leukemia	17 (43%)	10 (33%)	14 (47%)
Lymphoma	6 (15%)	9 (30%)	9 (30%)
Solid tumor	11 (28%)	8 (27%)	4 (13%)
Brain tumor	3 (8%)	0 (0%)	1 (3%)
Other	3 (8%)	3 (10%)	2 (7%)
Years from Diagnosis, Median (Range)	0.5 (0.0–11.4)	0.3 (0.0–9.9)	0.3 (0.0–11.3)
On Active Treatment, n (%)	34 (85%)	27 (90%)	26 (87%)
Hematopoietic Stem Cell Transplant, n (%)	8 (20%)	2 (7%)	4 (13%)
Inpatient at Interview, n (%)	13 (33%)	14 (47%)	16 (53%)

### SPARK family member web pages

Table 2 shows the results of the SPARK family member web pages evaluation and the number that were incorrect in their understanding of specific sections. Initially, there was no SPARK Family Member Home Page but after the first five participants, this need was identified and the page was added. Changes based upon participant feedback included simplifying language, adding bullet points and esthetic alterations. After 40 interviews, the SPARK family member web pages were considered satisfactory. That is, fewer than two interviewees were incorrect in their understanding of the web pages. In evaluating the website overall, none of the participants said that the SPARK family member web pages were hard or very hard to use.

**Table 2:** Understanding of SPARK family member web pages stratified by cohort^a,b^

SPARK Family Member Web Pages: Section and Subsections	Cohort 1 (*n* = 10)	Cohort 2 (*n* = 10)	Cohort 3 (*n* = 10)	Cohort 4 (*n* = 10)
SPARK Home Page				
Overall	0	0	0	0
What are SPARK and SSPedi?	0	0	0	1
Family Member Home Page				
Overall	0/5^c^	1	0	0
What can you do on this page?	1/5^c^	1	0	0
How would you see your child’s symptom scores?	1	0	0	0
Single SSPedi Administration Report (shows degree of bother for all 15 symptoms)				
Overall	0	0	0	0
Interpret degree of bother for a specific symptom	0	0	0	0
Specific Symptom Longitudinal Report (shows one symptom over time on a line graph)				
Navigate to specific symptom over time report	0	0	0	0
Interpret specific symptom over time	0	0	0	0
Interpret degree of bother on a specific date	0	0	0	0
Care Recommendations Landing Page				
Overall	3	0	0	0

Abbreviations: *SPARK* Supportive care Prioritization, Assessment and Recommendations for Kids; *SSPedi* Symptom Screening in Pediatrics Tool

^a^ Flow of interview described in text and in Additional file 1, which show evaluated sections

^b^ Understanding of each section rated by two interviewers on a 4-point Likert scale ranging from 1=” completely incorrect” to 4 = “completely correct”. The number of participants who were rated as completely or mostly incorrect are shown

^c^ Five participants were interviewed in this cohort as the need to test additional aspects of the web pages was identified and the interview structure was revised thereafter

### Family member care recommendations

Table 3 summarizes the results of the fatigue and mucositis family member care recommendations evaluation. Modifications included adding a definition of fatigue, edits to language to improve readability and addition of bullet points. Both family member care recommendation required three iterations of 10 participants each, or 30 participants total, to achieve finalization. Among the last 10 participants, none said that either family member care recommendation was hard to understand and none were incorrect in their understanding of the care recommendation sections. All final family member care recommendations may be found at: https://www.sungresearch.com/family-care-recommendations.

**Table 3:** Understanding of two family member care recommendations: Managing Fatigue and Preventing Mouth Sores^a,b^

Care Recommendations: Topics and Subsections	Cohort 1 (*n* = 10)		Cohort 2 (*n* = 10)		Cohort 3 (*n* = 10)	
Managing Fatigue	Hard^a^	Incorrect^b^	Hard^a^	Incorrect^b^	Hard^a^	Incorrect^b^
What are the recommended approaches to help my child manage their fatigue?	0	0	0	0	0	0
What else may help my child with their fatigue?	0	0	0	0	0	0
Is there any approach that should be avoided?	2	1	0	0	0	0
**Preventing Mouth Sores**						
Factors affecting your child’s management	0	1	0	0	0	0
Cryotherapy recommendation	0	1	0	0	0	0
Low-light therapy recommendation	0	0	0	0	0	0
Keratinocyte growth factor recommendation	0	0	0	0	0	0

^a^ How hard or easy was each section to understand as rated by participants. The number who rated the section as hard or very hard to understand is shown

^b^ Participant understanding of each section as rated by two clinical research associates. The number who were rated as mostly or completely incorrect is shown

## Discussion

In this study including family members of children and adolescents receiving cancer treatments, we successfully developed SPARK family member web pages accessing proxy-SSPedi and care recommendations for symptom management based on CPGs. We also developed a method of creating family member care recommendations based upon CPGs designed for healthcare professionals and specifically created “Managing Fatigue” and “Preventing Mouth Sores” care recommendations.

We found that iterative modifications based on the opinions of the intended users, namely family members of pediatric patients receiving cancer treatments, resulted in materials that were useable and understandable. Our results are consistent with other studies that performed user-testing to improve the understandability of a patient information sheet or an informed consent form using an iterative process [19–21]. Similar to our study, changes such as adding bullet points and changing language improved the documents understandability in subsequent rounds of testing [19–21]. While we cannot be certain that the cognitive interviews substantially improved SPARK web pages and care recommendations, the favorable results in the final cohorts suggest the usefulness of the approach.

As with any developed product, usability and understandability are prerequisites but do not necessarily translate to clinical usefulness. Future research should evaluate how SPARK family member web pages impacts on family and child outcomes.

A strength of this study was the large number of family member participants from various backgrounds, education levels and time since their child’s cancer diagnosis. Interviews were also conducted with two research assistants allowing for one to lead the interview and engage with the participant and the other to take field notes. Importantly, our methods incorporated two independent assessments of participant understanding of each element. Finally, our results are particularly encouraging since not all participants had English as a first language. However, this study is limited by its conduct at a single pediatric center. In addition, we only tested two family member care recommendations in this study. Understanding may vary with the CPG topic. Thus, it would be helpful to perform testing at more centers and with other CPG topics, particularly those involving more complex symptoms.

## Conclusion

In conclusion, we successfully developed SPARK web pages for use by family members of pediatric patients receiving cancer treatments. We also developed a process for translating CPG recommendations designed for healthcare professionals to lay language. The utility of the SPARK family member web pages after clinical implementation could be a focus for future research.

## Supplementary information

**Additional file 1.** SPARK family member web pages (Final versions)

## Data Availability

The datasets used and/or analysed during the current study are available from the corresponding author on reasonable request.

## Abbreviations

CPG: Clinical practice guideline
HSCT: Hematopoietic stem cell transplantation
SPARK: Supportive care Prioritization, Assessment and Recommendations for Kids
SSPedi: Symptom Screening in Pediatrics Tool
