# Supplementary material for: Development of the SPARK family member web pages to improve symptom management for pediatric patients receiving cancer treatments
Source: BMC Cancer. 2020 Sep 25;20:923. doi: 10.1186/s12885-020-07433-9 (PMC7519510; doi:10.1186/s12885-020-07433-9)
Supplement: Supplementary file 1 — Additional file 1. SPARK family member web pages (Final versions) [file 12885_2020_7433_MOESM1_ESM.pdf]

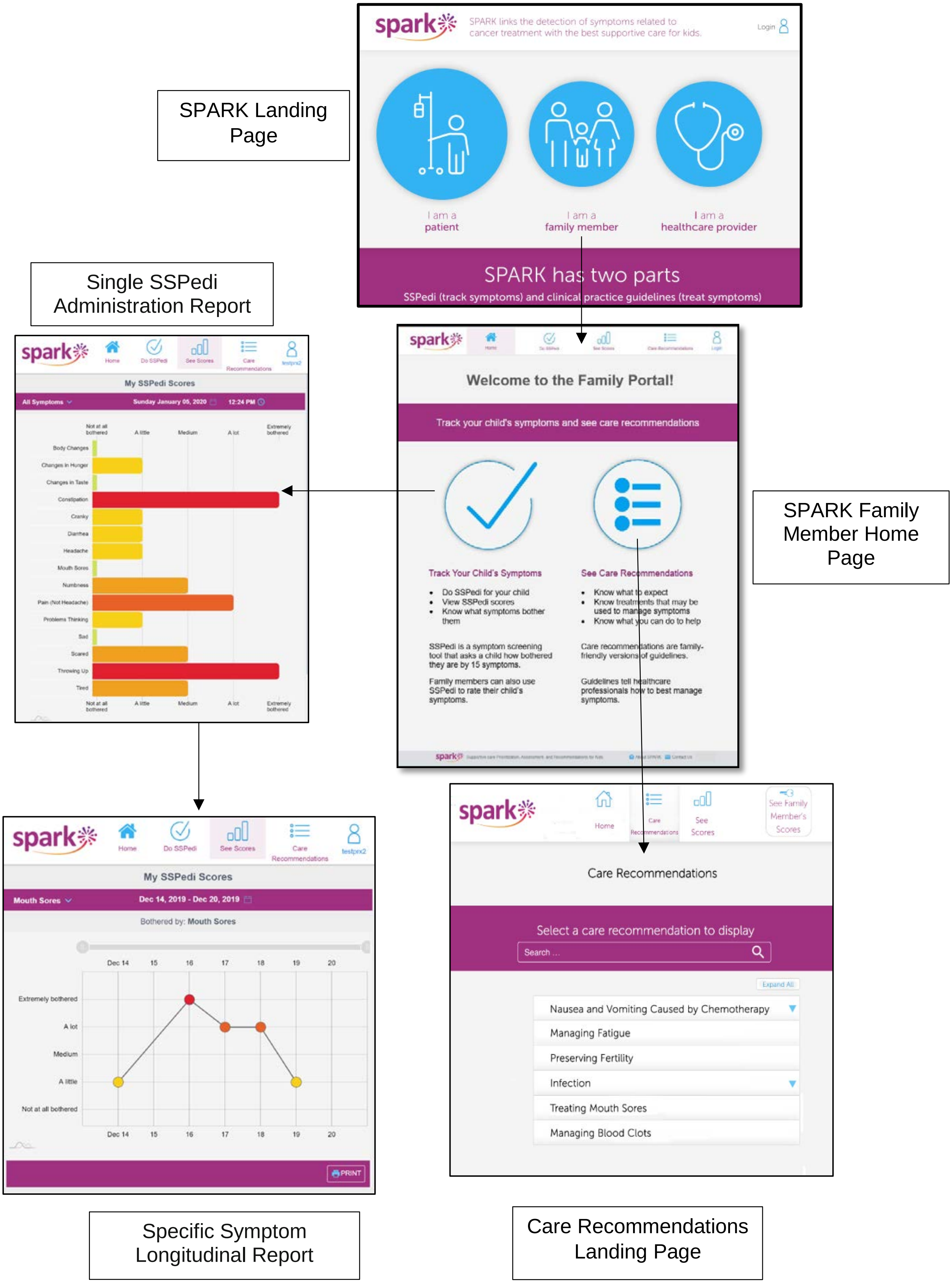
SPARK Landing Page
spark
SPARK links the detection of symptoms related to cancer treatment with the best supportive care for kids.
Login
I am a patient
I am a family member
I am a healthcare provider
SPARK has two parts
SSPedi (track symptoms) and clinical practice guidelines (treat symptoms)
Single SSPedi Administration Report
Home
Do SSPedi
See Scores
Care Recommendations
testprx2
My SSPedi Scores
All Symptoms
Sunday January 05, 2020
12:24 PM
Not at all bothered
A little
Medium
A lot
Extremely bothered
Body Changes
Changes in Hunger
Changes in Taste
Constipation
Cranky
Diarrhea
Headache
Mouth Sores
Numbness
Pain (Not Headache)
Problems Thinking
Sad
Scared
Throwing Up
Tired
SPARK Family Member Home Page
Welcome to the Family Portal!
Track your child's symptoms and see care recommendations
Track Your Child's Symptoms
Do SSPedi for your child
View SSPedi scores
Know what symptoms bother them
SSPedi is a symptom screening tool that asks a child how bothered they are by 15 symptoms.
Family members can also use SSPedi to rate their child's symptoms.
See Care Recommendations
Know what to expect
Know treatments that may be used to manage symptoms
Know what you can do to help
Care recommendations are family-friendly versions of guidelines.
Guidelines tell healthcare professionals how to best manage symptoms.
Specific Symptom Longitudinal Report
My SSPedi Scores
Mouth Sores
Dec 14, 2019 - Dec 20, 2019
Bothered by: Mouth Sores
Dec 14
15
16
17
18
19
20
Extremely bothered
A lot
Medium
A little
Not at all bothered
PRINT
Care Recommendations Landing Page
Home
Care Recommendations
See Scores
See Family Member's Scores
Care Recommendations
Select a care recommendation to display
Search ...
Expand All
Nausea and Vomiting Caused by Chemotherapy
Managing Fatigue
Preserving Fertility
Infection
Treating Mouth Sores
Managing Blood Clots
